# Betanin from *Beta vulgaris* Attenuates Complete Freund’s Adjuvant-Induced Inflammatory Pain: Integrated Preclinical and In Silico Insights

**DOI:** 10.3390/biomedicines14061202

**Published:** 2026-05-27

**Authors:** Ahmed Massoud, Amina E. Essawy, Mohammed A. Alfredan, Ashraf M. Abdel-Moneim, Rehab A. Gomaa, Sherine Abdel Salam

**Affiliations:** 1 Department of Zoology, Faculty of Science, Alexandria University, Alexandria 21511, Egypt; amina.essawy@alexu.edu.eg (A.E.E.); ashraf.abdelmoneim@alexu.edu.eg (A.M.A.-M.); rehab.hussein@alexu.edu.eg (R.A.G.); 2 Faculty of Science, Alamein International University, New Alamein City 51718, Egypt; 3 Department of Biological Sciences, Faculty of Science, King Faisal University, Al-Ahsa 31982, Saudi Arabia; malfredan@kfu.edu.sa

**Keywords:** betanin, pain model, neuroinflammation, miRNA-targeted therapy, molecular docking

## Abstract

**Background/Objectives**: Betanin (BET), a prominent phytochemical mainly derived from *Beta vulgaris*, exhibits strong anti-inflammatory and antioxidant activities owing to its distinctive chemical structure. Nevertheless, its potential analgesic effect in the context of inflammatory pain remains insufficiently explored. Accordingly, this study investigated the analgesic effects of BET in a complete Freund’s adjuvant (CFA)-induced rat model of inflammatory pain. **Methods**: Rats received a single subcutaneous injection of 100 µL CFA to induce inflammatory pain, followed by oral administration of BET at doses of 40 or 80 mg/kg/day for 14 days. **Results**: BET treatment significantly reduced paw edema and improved HPL (hot plate latency) in CFA-injected rats. Biochemically, in the ipsilateral spinal cord of rats, BET at both 40 and 80 mg/kg significantly increased IL-4, and only the 80 mg/kg dose significantly reduced oxidative stress (MDA) and IL-1β. TNF-α levels were slightly reduced at both doses and did not reach statistical significance versus CFA. At the molecular level, miR-107 was significantly downregulated by BET at 80 mg/kg (but not 40 mg/kg), while miR-145 was significantly upregulated by both 40 mg/kg and 80 mg/kg compared to CFA. Pearson’s correlation indicated that miR-107 was positively correlated with MDA, IL-1β and TNF-α but negatively with IL-4, whereas miR-145 was positively correlated with IL-4 but negatively with IL-1β. PCA biplot analysis corroborated these findings, showing simultaneous presence of MDA, IL-1β, TNF-α, and miR-107 with CFA, and IL-4 and miR-145 were only related to control and CFA+BET80 groups. In addition, using transmission electron microscopy imaging, we found that BET alleviated neuronal damage in CFA-treated rats. Furthermore, molecular docking analysis predicted that BET may exhibit stable binding interactions with several inflammation- and apoptosis-related targets, including AKT1, mTOR, IKKβ, TNF-α, IL-1β, COX-2, caspase-3, caspase-7, and caspase-8, supporting its multi-target anti-inflammatory and antiapoptotic effects. **Conclusions**: Overall, our data suggest that BET can possibly exert analgesic effects in CFA-induced inflammatory pain by modulating oxidative stress and favoring a shift toward an anti-inflammatory status. These effects coincided with downregulation of miR-107, overexpression of miR-145, and improvements in inflammatory pain behaviors. Further investigations are required to validate the involvement of specific miRNA- and pathway-mediated effects. Nevertheless, our findings highlight BET as a promising natural candidate for future development of anti-inflammatory and analgesic strategies.

## 1. Introduction

Pain is defined by the International Association for the Study of Pain (IASP) as an unpleasant sensory and emotional experience associated with or resembling that associated with actual or potential tissue damage [1]. Among the various types of pain, inflammatory pain is characterized by injury or damage of peripheral tissues along with localized inflammation in the damaged area [2]. Inflammation by itself is a protective physiological process that is driven by the release of multiple pro-inflammatory cytokines, such as IL-1β and TNF-α, as well as chemokines and other mediators from the inflamed tissues [3]. These mediators activate pain receptors at the site of injury, initiating the transfer of pain signals to the spinal cord and subsequently to the brain [4,5].

Emerging research suggests that, beyond traditional pain mediators, specific microRNAs (miRNAs) play a pivotal role in modulating inflammation and pain signaling [6,7]. MiRNAs are non-protein-coding RNA molecules (20–22 nucleotides) that regulate cellular gene expression at the post-transcriptional level [8]. Importantly, accumulating evidence indicates that miRNAs are not limited to inflammatory regulation but also act as key modulators of neuronal function, contributing to neuronal plasticity, synaptic regulation, and pain signal processing within the nervous system [9]. In this context, miRNAs have been shown to regulate genes involved in neuronal excitability, survival pathways, and nociceptive transmission, thereby influencing pain perception beyond classical inflammatory pathways [10]. Furthermore, miRNAs are increasingly recognized as critical contributors to central and peripheral sensitization, where they regulate glial activation, synaptic plasticity, and long-term changes in nociceptive circuits that underlie chronic pain conditions [11]. This broader regulatory role positions miRNAs as integrative molecular links between neuroinflammation, neuronal dysfunction, and pain chronification.

Among them, miR-107, which is consistently upregulated in the lumbar spinal dorsal horn of rats with CFA-induced inflammatory pain, where it contributes to pain maintenance by reducing inhibitory transmission, facilitating central sensitization, and sustaining the inflammatory pain state [12]. Conversely, miR-145 has been reported to exert analgesic effects in neuropathic pain models, and administration of miR-145 mimics alleviated both mechanical allodynia and thermal hyperalgesia in chronic constriction injury (CCI) rats, and suppressed pro-inflammatory cytokines such as TNF-α, IL-1β, and interleukin-6 (IL-6) [13]. Moreover, both miR-107 and miR-145 have been implicated in the regulation of oxidative stress pathways. For instance, inhibition of miR-107 activates AMPK–Nrf2 signaling, which reduces oxidative injury [14]. Additionally, miR-145 has been shown to attenuate oxidative stress and inflammatory responses by negatively regulating ARF6, reducing reactive oxygen species (ROS) levels and lipid peroxidation (LPO) [15]. Increasing evidence highlights the crucial role of oxidative stress in inflammatory pain, where elevated ROS and weakened antioxidant defenses sensitize nociceptive pathways and amplify neuroinflammation [16].

Conventional drugs used for the management of inflammatory pain predominantly include nonsteroidal anti-inflammatory drugs (NSAIDs), corticosteroids, and opioid analgesics [17,18]. Nevertheless, the clinical practice of these therapeutics is constrained by their associated side effects, ineffectiveness, and potential long-term complications [19,20,21]. Hence, the identification of safe, efficacious, and naturally derived alternatives for the mitigation of inflammatory pain remains a critical area of research. Indeed, recent comprehensive reviews highlight that naturally occurring compounds can effectively modulate complex interleukin-mediated networks, such as those involving IL-1β, IL-6 and IL-4, offering effective therapeutic options for chronic inflammatory conditions with potentially fewer adverse effects than synthetic drugs [22]. BET (betanidin 5-O-β-d-glucoside) is a water-soluble red pigment and bioactive compound that is found in the roots of Beetroot (*Beta vulgaris*) [23]. BET has garnered considerable scientific interest due to its putative possible health-promoting properties. Experimental studies have demonstrated that BET can attenuate ROS, reduce oxidative stress, and regulate gene activity [24,25]. Moreover, it exerts a broad spectrum of biological activities, including anti-inflammatory [26], anti-diabetic [27], anti-cancer [28], and neuro-protective effects [29]. However, despite its established therapeutic advantage and safety profile as a food additive (E162) [30], BET analgesic activity in a CFA-induced inflammatory pain model and its modulatory impact on inflammation-related cytokines and miRNAs in the LSPC have not yet been elucidated.

CFA, an oil suspension containing heat-killed Mycobacterium tuberculosis, is widely used to induce inflammatory responses in various rodent pain models, typically through subcutaneous injection into the paw [31]. Administration of CFA in the paw area results in an increase in the levels of the inflammatory mediators, such as TNF-α and IL-1β on the ipsilateral side. These cytokines play an important role in the progression of inflammation, which subsequently propagates to contralateral sites [32]. Multiple studies have shown that CFA-induced inflammation results in allodynia and hyperalgesia that appear within hours of injection and persists for at least two weeks; hence, CFA is usually used as a standard model to investigate inflammatory pain mechanisms [33,34,35]. Despite this extensive background, the application of BET as a therapeutic strategy for inflammatory pain has not yet been investigated. Therefore, this study seeks to investigate the analgesic efficacy of BET in a CFA-induced inflammatory pain model by combining biochemical and molecular analyses with in silico molecular docking. This integrative approach provides a novel framework that bridges experimental outcomes with computational predictions, hence offering new perspectives on BET’s potential role in pain modulation.

## 2. Materials and Methods

### 2.1. Animals and Chemicals

Twenty-four adult male Wistar albino rats (weighing 160–180 g) were procured from the animal research facility at Alexandria University, Alexandria, Egypt. Animals were housed in polypropylene cages with three rats per cage, maintained under controlled environmental conditions: a temperature of 22 ± 2 °C, relative humidity of 50–60%, and a 12:12 light-dark cycle. Rats were provided with a standard laboratory diet and free access to water ad libitum throughout the study. A one-week acclimatization period was allowed prior to the start of experiments to facilitate adaptation to the laboratory conditions.

CFA, containing heat-killed *Mycobacterium tuberculosis* (strain H37Ra, ATCC 25177) suspended in paraffin oil at a concentration of 1 mg/mL, was obtained from Sigma–Aldrich (St. Louis, MO, USA; Catalog number F5881). BET, derived from red beet extract and diluted with dextrin (CAS number 7659-95-2), was also sourced from Sigma–Aldrich (Catalog number 901266). Colorimetric assay kit for determination of MDA was supplied by Biodiagnostic Company (Giza, Egypt). Enzyme-linked immunosorbent assay (ELISA) kits for the quantification of IL-4 (Cat. Number ELK1154), IL-1β (Cat. Number ELK1272), and TNF-α (Cat. Number ELK1396) were purchased from ELK Biotechnology Co., Ltd. (Wuhan, China). For quantitative reverse transcription–polymerase chain reaction (qRT–qPCR) analyses, the miRCURY LNA miRNA PCR Starter Kit (Catalog number 339320) was procured from Qiagen (Hilden, Germany).

### 2.2. Experimental Design

**Figure 1:**
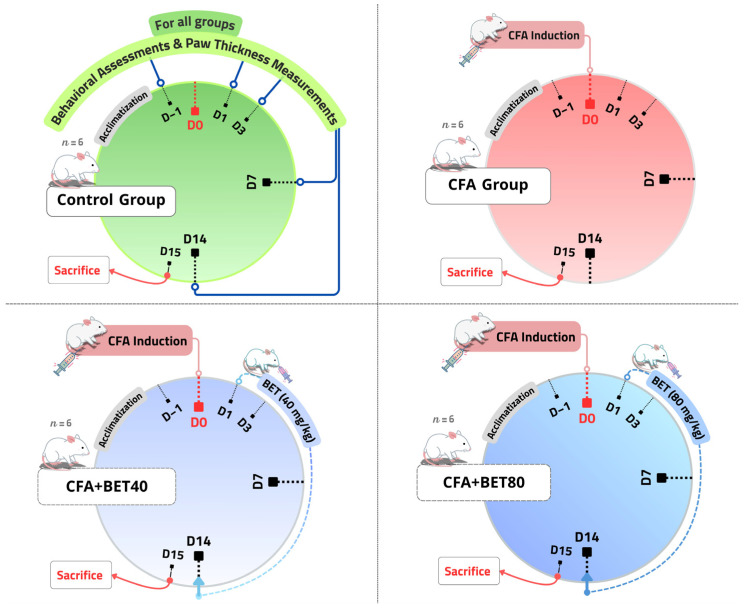
Schematic representation of the experimental timeline and treatment schedule employed in the study.

Rats were randomly divided into four experimental groups (*n* = 6 per group). The sample size was determined based on established protocols from previous similar studies [36,37]. Animals were treated as follows: (1) Control group: received neither CFA injection nor any treatment; (2) CFA group: in which 100 µL of CFA was subcutaneously injected into the plantar surface of the right hind paw to induce inflammatory pain [35]; (3) CFA + BET40 group: received an oral dose of BET (40 mg/kg/day) starting one day after CFA injection and continued for 14 consecutive days; and (4) CFA + BET80 group: in which BET was administered orally at a dose of 80 mg/kg/day under the same schedule (Figure 1). The selected BET doses were based on cumulative dose ranges reported in the prior literature [27,38]. Paw thickness, as an index of peripheral inflammation, was measured using a digital thickness gauge (Dasqua 0–25 mm/0–1, Italy) one day prior to CFA injection (day −1; baseline), and subsequently on days 1, 3, 7, and 14 post CFA-injection. Behavioral (thermal) hyperalgesia test was conducted on the same days following a 30 min habituation period to the testing environment. The same cohort of animals (*n* = 6) was evaluated at all time points for paw thickness and behavioral outcomes. Throughout the duration of the experiment, no adverse health effects, morbidity, or unplanned mortalities were encountered in any of the treatment or control groups. On day 15, rats were euthanized via an overdose of isoflurane anesthesia followed by decapitation. The ipsilateral segment of the LSPC, specifically the L4–L6 segments, was rapidly isolated by hydraulic extrusion [39]. Following euthanasia, three rats per group were allocated for biochemical and molecular assessments, while the remaining three were used for transmission electron microscopy (TEM) examination after being cardially perfused with buffered 4F1G (4% formaldehyde and 1% glutaraldehyde). To avoid bias, all evaluation were performed in a blinded manner.

### 2.3. Thermal Pain Sensitivity Test

To assess thermal hyperalgesia, the HPL was measured using a hot plate test, following the methodology adapted from Yin et al. [40]. Each rat was individually placed on a heated metal surface maintained at 50 °C or 52 °C. Care was taken to ensure that both hind paws made gentle contact with the plate surface without applying any external pressure. A cutoff time of 30 s was established to prevent or minimize the risk of tissue damage. The thermal pain threshold was determined by recording the latency to the first nociceptive response, characterized by paw shaking, withdrawal, or licking behavior.

### 2.4. Biochemical Parameters

A portion of ipsilateral LSPC was perfused with phosphate-buffered saline (PBS; pH 7.4) containing 0.16 mg/mL heparin to eliminate blood clots and residual erythrocytes. Following perfusion, the tissue was homogenized in an ice-cold buffer composed of 50 mM potassium phosphate (pH 7.4) and 1 mM EDTA. The homogenate was subsequently centrifuged at 4000 rpm for 15 min at 4 °C, and the resulting supernatant was collected and stored for subsequent biochemical analyses.

MDA levels were determined, based on the thiobarbituric acid reactive substances (TBARS) assay, as described by Satoh [41] and Ohkawa et al. [42]. In this test, MDA reacts with thiobarbituric acid (TBA) at 95 °C for 30 min to produce a pink chromogen that was detected spectrophotometrically at 534 nm. The levels of anti-inflammatory (IL-4) and pro-inflammatory (IL-1β and TNF-α) cytokines in the LSPC were measured using ELISA kits in accordance with the guidelines provided by the manufacturers. Each micro-ELISA strip plate was pre-coated with a specific capture antibody. Standards or tissue samples were added to the wells and allowed to bind to the immobilized antibody. After washing the wells, an HRP-conjugated detection antibody specific to IL-4, IL-1β, or TNF-α was added. Colorimetric detection was achieved through substrate addition, which produced a blue color that changed to yellow after adding the stop solution. Absorbance at 450 nm was measured spectrophotometrically, and the cytokine concentrations were expressed as pg/mg protein. A standard calibration curve (0–1000 pg/mL) was generated for each assay. The intra-assay and inter-assay coefficients of variation reported by the manufacturer were <8% and <10%, respectively. Total protein concentration was determined by measuring absorbance at 280 nm using a NanoDrop 2000 spectrophotometer (Thermo Scientific, Waltham, MA, USA) [43].

### 2.5. MicroRNA Extraction and Quantitative Real-Time PCR (qRT–PCR) Assay

Total RNA, including miRNA, was extracted from a separate portion of ipsilateral LSPC using the QIAzol-based miRNeasy Mini Kit (Qiagen, Germany) through following the manufacturer’s protocol. The concentration and purity of the isolated RNA were assessed using a NanoDrop 2000 spectrophotometer (Thermo Scientific, USA) by measuring absorbance at 260 nm. The obtained A260/A280 ratio ranged from (2.05–2.11). Reverse transcription of miRNA into complementary DNA (cDNA) was carried out using the miRCURY LNA miRNA PCR Starter Kit (Qiagen, Germany) for qRT-PCR following the manufacturer’s protocol. Amplification was performed on a Rotor-Gene^®^ real-time PCR system (Qiagen, Germany), which does not require ROX as a reference dye. Primers specific to miR-107 and miR-145, along with a candidate endogenous control (miR-103-3p), were included in the kit synthesized by Qiagen and their assay IDs are included in Table 1. The amplification curves were monitored, cycle threshold (Ct) values were obtained, and relative expression levels were calculated using the 2^−ΔΔCT^ method [44].

### 2.6. Transmission Electron Microscopy

The right dorsal horn of the LSPC was excised and immersed in a fixative containing 4F1G in phosphate buffer of pH 7.2 for 3 h at 4 °C to prepare samples for TEM. Following fixation, the tissues were post-fixed with 2% osmium tetroxide for 2 h at 4 °C, rinsed in buffer, dehydrated through a graded ethanol series, and embedded in epon–araldite resin. Ultrathin sections (~50 nm) were then cut, placed on copper grids, contrasted with uranyl acetate and lead citrate, and analyzed using a JEOL JEM-1400Plus TEM (JEOL Ltd., Tokyo, Japan). Ultrastructural observations were qualitatively assessed using representative fields from ipsilateral LSPC sections of each experimental group.

### 2.7. Molecular Docking Analysis

In silico molecular docking simulations were conducted to evaluate potential binding interactions between BET (CAS: 7659-95-2, retrieved from PubChem) and key inflammation- and apoptosis-related targets (AKT1, mTOR, IKKβ, TNF-α, IL-1β, COX-2, caspase-3, caspase-7, and caspase-8). Three-dimensional crystal structures of all targets were retrieved from the Protein Data Bank (PDB). Where necessary, missing residues were modeled using MODELLER via UCSF ChimeraX 1.9 [45,46] to ensure structurally complete models. Molecular docking was performed using AutoDock Vina version 1.2.3 which employs a stochastic global optimization algorithm combined with iterated local search and BFGS-based local minimization [47,48]. The docking protocol was validated by redocking the native ligands into their respective active sites, with root-mean-square deviation (RMSD) values below 2.0 Å considered indicative of a reliable procedure. Following docking, the resulting binding poses and molecular interactions were visualized and analyzed using BIOVIA Discovery Studio 2024. Complete details regarding PDB IDs, protein and ligand preparation protocols, charge assignments, and specific grid box dimensions are provided in Supplementary Method S1 and Supplementary Table S1.

### 2.8. Statistical Analyses

Statistical analyses were conducted using SPSS software program for Windows (version 16.0). Data were presented as mean ± standard error (SE). The normality of distributions was assessed via the Shapiro–Wilk test, while the homogeneity of variances was evaluated using Levene’s test. The distributions of variables were also checked using the Skewness and Kurtosis coefficients and q–q plots. For time-dependent datasets (paw thickness and HPL), results were analyzed using Friedman’s test, which is a nonparametric repeated-measures ANOVA. Kendall’s W served as the effect size index for the Friedman’s test. The level of concordance was interpreted using the following categories: no agreement (W < 0.10), weak agreement (0.10 ≥ W < 0.30), moderate agreement (0.30 ≥ W < 0.60), strong agreement (0.60 ≥ W < 1.0), and perfect agreement (W = 1) [49]. In addition, Kruskal–Wallis test and Mann–Whitney U test were used to evaluate differences at each time point. For other data, one-way analysis of variance (ANOVA) was applied to determine significant differences among groups, with subsequent pairwise comparisons performed using the least significant difference (LSD) post hoc test. Partial eta squared (η^2^) was used to estimate the effect size for one-way ANOVA, with thresholds of ≥0.01 for small, ≥0.06 for medium, and ≥0.14 for large effects [50]. Pearson’s correlation coefficient (r) was employed by means of the Pearson’s correlation method to assess relationships between variables. Furthermore, Principal Component Analysis (PCA) was carried out using Minitab 17.0. Statistical significance was set at *p* ≤ 0.05.

## 3. Results

### 3.1. BET Reduces the Development of CFA-Induced Paw Edema in Rats

**Figure 2:**
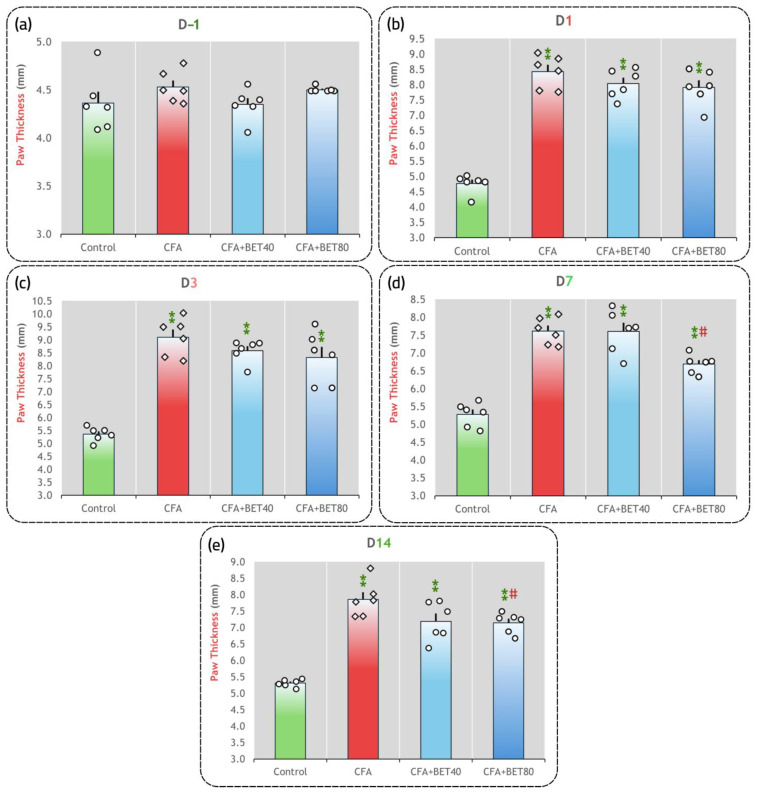
Effects of CFA-induced inflammation and BET treatment on paw thickness in male rats on day −1 (**a**), day 1 (**b**), day 3 (**c**), day 7 (**d**), and day 14 (**e**). Data are presented as mean ± SE (*n* = 6, individual data points shown). Significance was determined by Mann–Whitney U test. ⁑ *p* < 0.05 vs. control; # *p* < 0.05 vs. CFA.

Friedman’s test indicated a significant time effect on paw swelling [χ^2^_(4)_ = 64.894, *p* < 0.001, Kendall’s W = 0.676]. As shown in Figure 2a, at baseline (day −1), no significant differences in paw thickness among the experimental groups, demonstrating that all animals started from comparable physiological conditions prior to CFA induction or treatment [χ^2^_(3)_ = 6.722, *p* = 0.081]. Following CFA injection, a marked increase in paw edema was observed on day 1 in the CFA, CFA+BET40, and CFA+BET80 groups compared to the control group (all *p* = 0.004), confirming successful induction of acute inflammation (Figure 2b). By day 3, the CFA group continued to exhibit significantly elevated paw thickness relative to the control (*p* = 0.004). Although BET treatment at both 40 mg/kg and 80 mg/kg resulted in a reduction in paw swelling compared to the CFA group, these effects did not reach statistical significance (*p* = 0.200 and *p* = 0.261, respectively) (Figure 2c). On day 7, paw edema remained significantly elevated in the CFA group compared to control (*p* = 0.004). BET at 40 mg/kg did not significantly alter paw thickness compared to the CFA group (*p* = 1.000), whereas BET at 80 mg/kg significantly reduced paw thickness (*p* = 0.004), demonstrating a dose-dependent effect (Figure 2d). By day 14, inflammation in the CFA group persisted at levels significantly higher than control (*p* = 0.004). In contrast, both BET treatment groups exhibited reductions in paw thickness compared to the CFA group. However, the reduction observed at 40 mg/kg did not reach statistical significance (*p* = 0.078), whereas the decrease at 80 mg/kg was statistically significant (*p* = 0.010). Despite these reductions, paw thickness in both BET-treated groups remained significantly higher than that of the control group (all *p* = 0.004) (Figure 2e).

### 3.2. BET Prevents Thermal Hyperalgesia Caused by the CFA Injection

**Figure 3:**
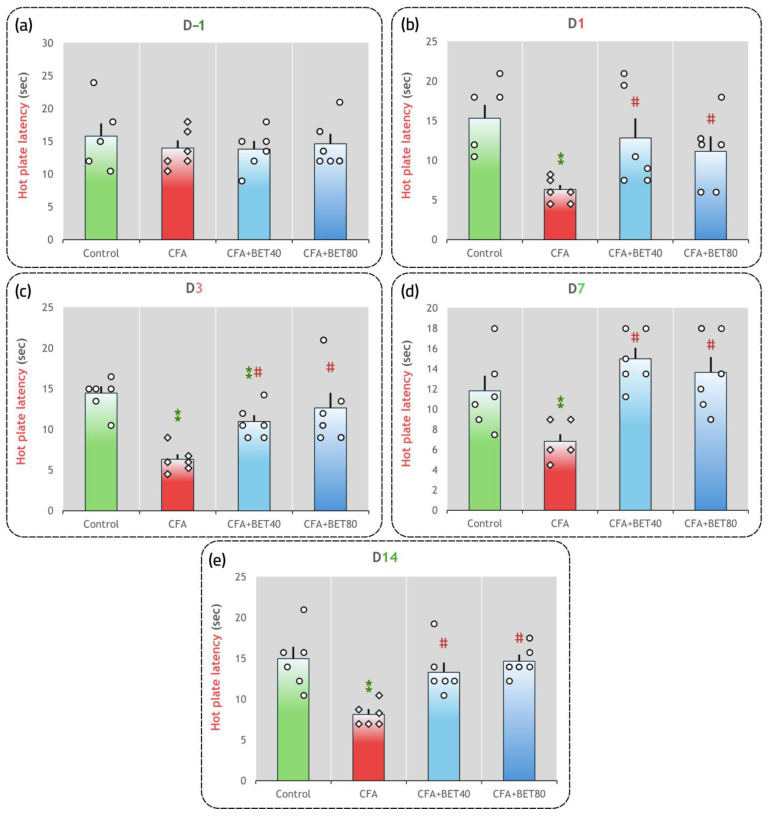
Effects of CFA-induced inflammation and BET treatment on thermal hyperalgesia in male rats on day −1 (**a**), day 1 (**b**), day 3 (**c**), day 7 (**d**), and day 14 (**e**). Data are presented as mean ± SE (*n* = 6, individual data points shown). Significance was determined by Mann–Whitney U test. ⁑ *p* < 0.05 vs. control; # *p* < 0.05 vs. CFA.

Friedman’s test conducted to assess thermal hyperalgesia by HPL across the different testing days revealed a significant main effect of time [χ^2^_(4)_ = 16.819, *p* = 0.002, Kendall’s W = 0.175]. At baseline (day −1), no significant differences in HPL were observed among the experimental groups [χ^2^_(3)_ = 0.581, *p* = 0.901], confirming comparable thermal sensitivity across all animals prior to the induction of inflammation or BET administration (Figure 3a). By day 1 post-CFA injection, Mann–Whitney U demonstrated a significant reduction in HPL in the CFA group relative to controls (*p* = 0.004), indicating the onset of thermal hyperalgesia. Notably, BET40 and BET80 significantly attenuated the reduction in latency compared to the CFA group (*p* = 0.009 and 0.049, respectively), implying an early antinociceptive effect that restored HPL toward normalcy (Figure 3b). On day 3, the CFA group continued to exhibit a marked decrease in HPL relative to the control group (*p* = 0.004). However, BET treatment at both 40 mg/kg and 80 mg/kg significantly increased HPL compared to the CFA group (all *p* = 0.006), with BET40 producing a partial improvement, whereas BET80 brought HPL close to normal levels (Figure 3c). By day 7, the CFA group maintained significantly reduced HPL values compared to controls (*p* = 0.015). BET administration at both dosages resulted in a significant increase in HPL relative to the CFA group (*p* < 0.004 for BET40 and *p* = 0.006 for BET80), achieving values comparable to controls and further supporting the sustained antinociceptive properties of BET (Figure 3d). On day 14, post hoc comparisons revealed that the CFA group still demonstrated significantly reduced HPL when compared to controls (*p* < 0.005). The BET-treated groups continued to show significant increases in HPL compared to the CFA group (*p* = 0.004 for BET40 and *p* = 0.003 for BET80), with their HPL levels reaching near-normal values (Figure 3e).

### 3.3. BET Decreases MDA Production and Inhibits Inflammatory Condition in CFA-Treated Rats

**Figure 4:**
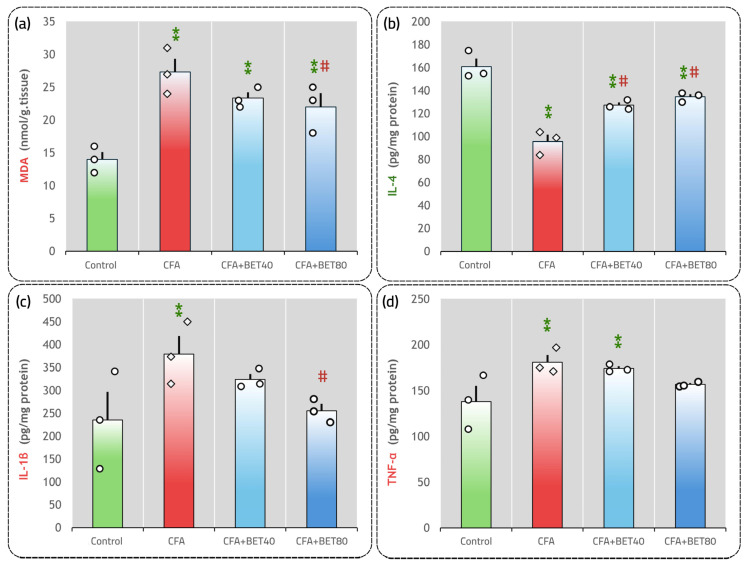
Effects of 14-day BET administration on oxidative stress marker MDA (**a**), anti-inflammatory cytokine IL-4 (**b**), and pro-inflammatory cytokines IL-1β (**c**) and TNF-α (**d**) in LSPC tissues of male rats with CFA-induced inflammatory pain. Data are presented as mean ± SE (*n* = 3, individual data points shown). Significance was determined by LSD post hoc test. ⁑ *p* < 0.05 vs. control; # *p* < 0.05 vs. CFA.

TBARS assay for MDA in the LSPC was used to assess oxidative damage to lipids. However, the TBARS method has certain restrictions. First, many of the free radicals detected are formed during the heating procedure, which can lead to artificially elevated values. Second, TBA lacks specificity for lipid-derived radicals, as it can also react with different oxidized compounds besides MDA. Therefore, instead of serving as a precise marker of LPO, TBARS is better regarded as a broad measure of tissue oxidation, rather than being a particular indicator of LPO [51]. Statistical evaluation using one-way ANOVA revealed significant variations in MDA levels among the experimental groups [F_(3,8)_ = 11.846, *p* = 0.003, η^2^ = 0.816, observed power = 0.980]. Subsequent LSD post hoc comparisons demonstrated a pronounced increase in MDA concentrations in the CFA group compared to controls (*p* < 0.001). Treatment with BET40 elicited a reduction in MDA levels relative to the CFA group, but this change did not reach statistical significance (*p* = 0.120). Notably, administration of BET80 significantly lowered MDA levels when compared to the CFA group (*p* = 0.049) (Figure 4a).

Levels of the anti-inflammatory cytokine IL-4 differed significantly across experimental groups, as identified by one-way ANOVA [F_(3,8)_ = 29.804, *p* < 0.001, η^2^ = 0.918, observed power = 1.000]. Post hoc analysis using the LSD test showed a marked reduction in IL-4 concentrations in the CFA group relative to the control group (*p* < 0.001), while treatment with BET at both 40 mg/kg and 80 mg/kg significantly elevated IL-4 levels compared to the CFA group (*p* = 0.002 and *p* < 0.001, respectively), as illustrated in Figure 4b.

Regarding proinflammatory cytokines, IL-1β exhibited a trend towards significance that did not meet the *p* ≤ 0.05 threshold [F_(3,8)_ = 3.054, *p* = 0.092, η^2^ = 0.534, observed power = 0.495]; however, pairwise comparisons revealed a significant elevation in the CFA group compared to controls (*p* = 0.027). In addition, treatment with BET at 40 mg/kg (CFA+BET40) led to a non-significant reduction in IL-1β levels relative to the CFA group (*p* = 0.326). While, BET administration at 80 mg/kg (CFA+BET80) statistically lowered IL-1β when compared to the CFA group (*p* = 0.049), with values comparable to the control group (*p* = 0.713), indicating a dose-dependent anti-inflammatory effect (Figure 4c). For TNF-α, the results showed that TNF-α levels approached a borderline statistical significance [F_(3,8)_ = 4.014, *p* = 0.051, η^2^ = 0.601, observed power = 0.617]; nevertheless, LSD post hoc group comparisons showed significantly higher TNF-α levels in the CFA group compared to controls (*p* = 0.013). Treatment with BET at 40 mg/kg (CFA+BET40) yielded a slight, yet statistically non-significant, reduction compared to the CFA group (*p* = 0.635). A further decrease in TNF-α levels was noted following administration of BET at 80 mg/kg (CFA+BET80), although this change did not achieve statistical significance (*p* = 0.113 vs. CFA) (Figure 4d).

### 3.4. Expression Profile of miR-107 and miR-145 in CFA and/or BET-Treated Rats

**Figure 5:**
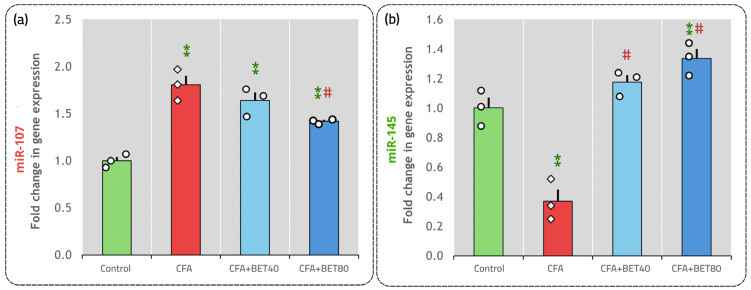
Effects of 14-day BET administration on the expression of miR-107 (**a**) and miR-145 (**b**) in LSPC tissues of male rats with CFA-induced inflammatory pain. Data are presented as mean ± SE (*n* = 3, individual data points shown). Significance was determined by LSD post hoc test. ⁑ *p* < 0.05 vs. control; # *p* < 0.05 vs. CFA.

According to the one-way ANOVA, there were significant variations in the expression of both miR-107 [F_(3,8)_ = 26.239, *p* ˂ 0.001, η^2^ = 0.908, observed power = 1.000] and miR-145 [F_(3,8)_ = 40.777, *p* ˂ 0.001, η^2^ = 0.939, observed power = 1.000] between and within the experimental groups. Post hoc LSD analysis indicated that miR-107 expression in the LSPC was significantly elevated in the CFA group relative to controls (*p* ˂ 0.001). While the CFA+BET40 group showed a non-significant reduction compared to CFA (*p* = 0.122), miR-107 levels were significantly downregulated in the CFA+BET80 group compared to CFA (*p* = 0.004), as shown in Figure 5a. Regarding miR-145, expression was markedly suppressed in the CFA group versus controls (*p* ˂ 0.001). Treatment with BET at both 40 mg/kg and 80 mg/kg significantly increased miR-145 levels compared to CFA, with both groups exhibiting significant upregulation (*p* ˂ 0.001) (Figure 5b).

### 3.5. Pearson’s r

For a more detailed understanding of miRNAs alterations, the study utilized Pearson’s correlation analysis to assess how miR-145 and miR-107 relate to the biochemical parameters. As seen in Table 2, a moderate negative correlation was observed between miR-145 and IL-1β (r = −0.634, *p* = 0.027), whereas miR-145 demonstrated a moderate positive correlation with IL-4 (r = 0.585, *p* = 0.045). On the other hand, miR-107 showed a strong positive correlation with MDA levels (r = 0.840, *p* = 0.001), along with moderate positive correlations with IL-1β (r = 0.591, *p* = 0.043) and TNF-α (r = 0.686, *p* = 0.014). Notably, miR-107 exhibited a strong inverse correlation with IL-4 (r = −0.908, *p* < 0.001).

### 3.6. Multivariate Pattern Using PCA

**Figure 6:**
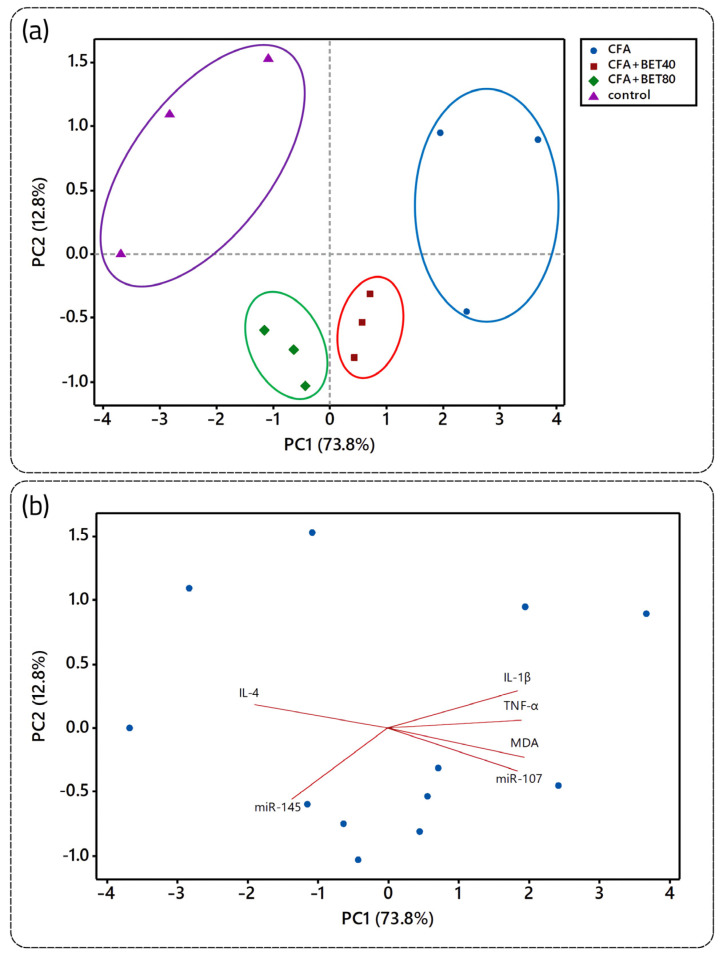
(**a**) Score plot: PCA score plot showing the clustering of the studied groups based on treatments. (**b**) Biplot: A biplot integrating the score plot and loading plot, providing contributions of variables to the group separation.

The data sets were then subjected to PCA to elucidate underlying variance patterns. The analysis revealed that the first principal component (PC1) accounted for the majority of the total variance (73.8%), whereas the second principal component (PC2) explained an additional 12.8%. The PCA score plot (Figure 6a) revealed distinct clustering of experimental groups, with control samples localized on the left and CFA samples positioned on the right. Furthermore, a progressive separation was observed among the CFA, CFA+BET40, and CFA+BET80 groups. Notably, the CFA+BET80 group exhibited a distribution profile closely aligned with that of the control group, suggesting a robust restorative effect of the higher BET dosage. Biplot analysis (Figure 6b) further supported these findings, with MDA, IL-1β, TNF-α, and miR-107 loading strongly toward the CFA cluster, whereas IL-4 and miR-145 were more closely associated with the control and CFA+BET80 groups.

### 3.7. Neuronal Lesions in CFA and/or BET-Treated Rats

**Figure 7:**
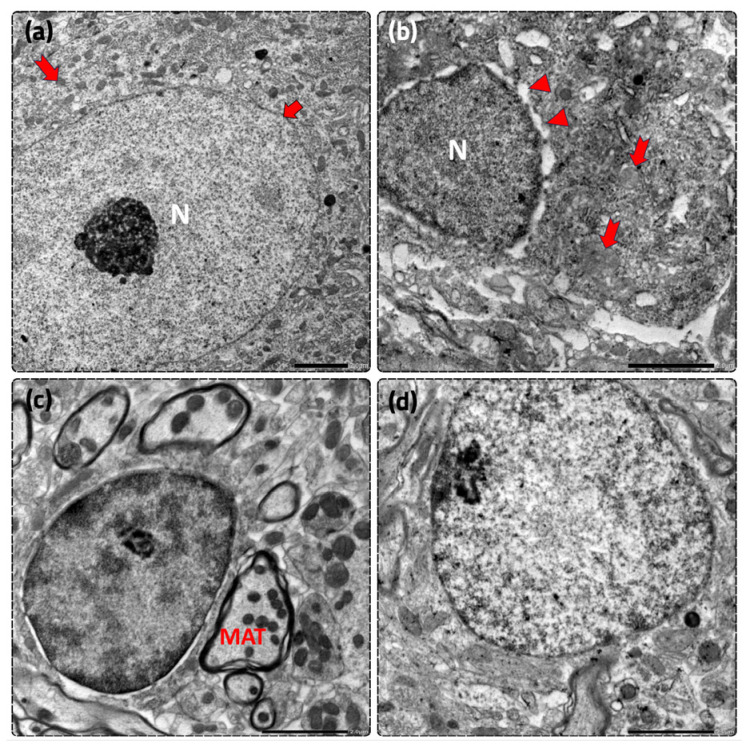
Representative transmission electron micrographs of ipsilateral LSPC neurons. (**a**) Control: observe normal ultrastructural features of neurons, including an intact nucleus (N), well-defined nuclear envelope (red arrow), and normal-looking mitochondria (forked arrow). (**b**) CFA: Marked neuronal disruption is discernible, characterized by shrinkage, chromatin condensation within the nucleus (N), membranous vesicle-appearing structures or blebs (arrowheads) in an expanded perinuclear space, and edematous/swollen mitochondria (forked arrow). (**c**) CFA+BET40: Mild neuronal lesions can be seen, indicating partial protection against CFA-induced injury. MAT = myelinated axon terminal. (**d**) CFA+BET80: Neuronal changes are minimal, reflecting pronounced neuroprotective effect. Bars = 2 μm.

In the control group, ultrastructural examination of the ipsilateral LSPC dorsal horn revealed neurons with preserved nuclear and cytoplasmic morphology, whereas CFA-treated animals exhibited noticeable neuronal ultrastructural alterations, including chromatin condensation (hyperchromatic nuclei), nuclear membrane irregularities such as blebbing, and swollen mitochondria, features commonly associated with cellular stress and apoptotic cell death. However, neuronal nuclei, nuclear envelopes, and mitochondrial configurations were mostly retained (mildly protected) in animals receiving BET at 40 mg/kg, with more prominent structural integrity was observed in the CFA+BET80 treatment group (Figure 7).

### 3.8. BET Interacts with AKT1, mTOR, IKKβ, TNF-α, IL-1β, COX-2; and Caspase-3, -7 and -8 in Molecular Docking Analysis

Docking analysis revealed that BET exhibited stable interactions with AKT1, mTOR Kinase, IKKβ, TNF-α, IL-1β, COX-2, caspase-3, caspase-7, and caspase-8. The binding affinities of BET and the corresponding co-crystallized ligands, along with RMSD values from redocking, are presented in Table 3. All RMSD values were below 2 Å, confirming the reliability of the docking protocol.

**Figure 8:**
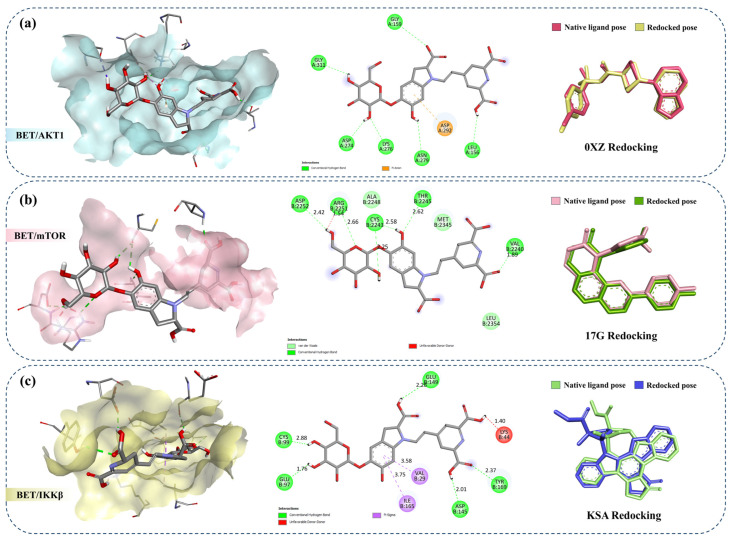
3D binding poses and 2D interaction diagrams of BET with (**a**) AKT1, (**b**) mTOR, and (**c**) IKKβ, alongside superimposed native and redocked co-crystallized ligands demonstrating docking validation.

BET established multiple interactions across all investigated targets, supporting the stability of the formed complexes. As illustrated in Figure 8a, within AKT1, BET formed six conventional hydrogen bonds with LEU156, GLY159, ASP274, LYS276, ASN279, and GLY311 (all in chain A), with bond distances ranging from 1.88 to 3.08 Å. Additionally, a π–anion interaction with ASP292 (chain A) further reinforced ligand binding. In mTOR (Figure 8b), BET established six hydrogen bonds with VAL2240, CYS2243, THR2245, ARG2251, and ASP2252 (chain B), with bond lengths ranging from 1.89 to 2.66 Å, indicating favorable polar interactions within the binding pocket. For IKKβ (Figure 8c), BET engaged in five hydrogen bonds with GLU97, CYS99, ASP145, GLU149, and TYR169 (chain B), with bond distances between 1.76 and 2.88 Å. In addition, π–sigma hydrophobic interactions with VAL29 and ILE165 (chain B) contributed to ligand accommodation within the active site.

**Figure 9:**
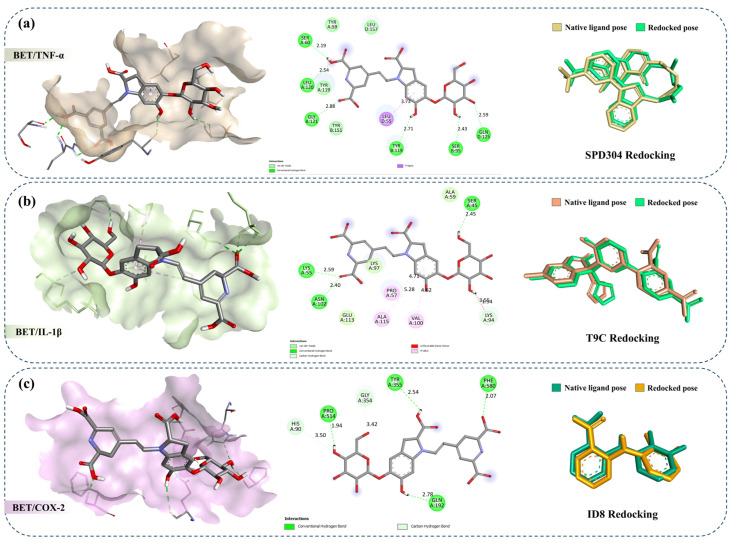
3D binding conformations and corresponding 2D interaction profiles of BET with (**a**) TNF-α, (**b**) IL-1β, and (**c**) COX-2, accompanied by the superimposition of native and redocked co-crystallized ligands to confirm the accuracy of the docking protocol.

In TNF-α (Figure 9a), BET formed six hydrogen bonds with SER60, LEU120, GLY121 (chain A), SER95, TYR119 (chain B), and GLN125 (chain D), with bond lengths ranging from 2.19 to 2.88 Å, along with a π–sigma interaction with LEU55 (chain D). Similarly, in IL-1β (Figure 9b), five hydrogen bonds were observed with SER45, LYS55, LYS94, and ASN102 (chain A), with bond distances of 2.40–3.55 Å. Additionally, three π–alkyl interactions involving PRO57, VAL100, and ALA115 (chain A) were identified, supporting ligand stabilization within the binding pocket. In COX-2 (Figure 9c), BET formed six hydrogen bonds with HIS90, HIS121, GLN192, GLY354, TYR355, PRO514, and PHE580 (chain A), with bond lengths ranging from 1.94 to 3.50 Å, indicating strong polar interactions within the active site.

**Figure 10:**
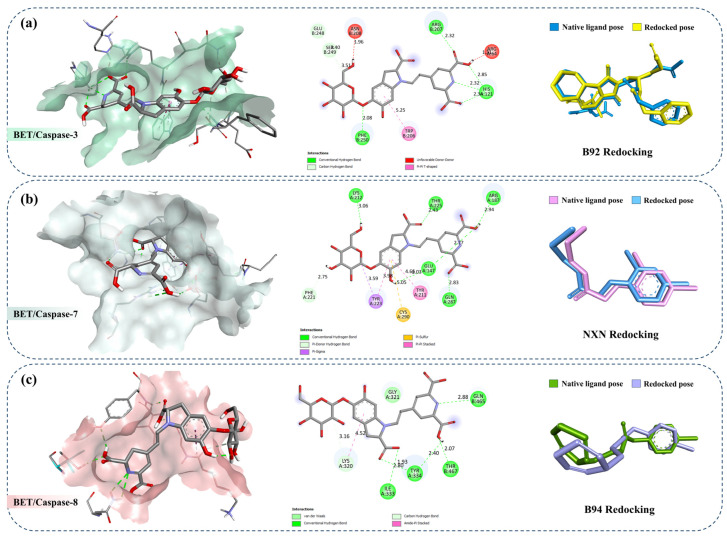
3D binding visualizations and corresponding 2D interaction representations of BET with (**a**) caspase-3, (**b**) caspase-7, and (**c**) caspase-8, accompanied by the superimposition of native and redocked co-crystallized ligands, demonstrating the robustness of the docking protocol.

Regarding apoptotic targets, BET interacted with caspase-3 (Figure 10a) through seven hydrogen bonds involving HIS121 (chain A), ARG207, GLU248, SER249, and PHE250 (chain B), with bond distances ranging from 2.08 to 3.40 Å. A π–π T-shaped interaction with TRP206 (chain B) was also observed. In caspase-7 (Figure 10b), BET formed nine hydrogen bonds with GLU147, ARG187, LYS212, PHE221, THR225, and GLN287 (chain A), with bond lengths between 2.00 and 3.06 Å. Additional hydrophobic interactions, including π–sigma, π–sulfur, and π–π stacking interactions with TYR211, TYR223, and CYS290 (chain A), further supported binding. Finally, in caspase-8 (Figure 10c), BET established six hydrogen bonds with LYS320, ILE333, TYR334 (chain A), GLN465, and THR467 (chain B), with bond distances ranging from 1.93 to 3.16 Å, alongside an amide–π stacked interaction involving LYS320 and GLY321 (chain A). A comprehensive overview of all ligand–receptor interactions is provided in Table 4.

## 4. Discussion

In this study, we investigated the analgesic potential of BET in a rat model of CFA-induced inflammatory pain. It is well established that CFA injection into the hind paw of rodents is used to induce and mimic persistent inflammatory pain in humans, making it the most prevalent model for investigating inflammatory pain etiology and mechanisms [52,53]. Our findings demonstrated that BET effectively alleviated paw edema produced by CFA-injection, indicating a pronounced anti-inflammatory effect, which is consistent with other researchers [54], who showed that betalain-rich *Beta vulgaris* dye significantly inhibited paw edema induced by CFA in mice. Moreover, BET increased HPL in rats treated with CFA, thus confirming its anti-nociceptive action, as reduced HPL is associated with increased pain sensitivity [55], and consistent with prior work showing thermal hyperalgesia in CFA models [56,57]. Numerous studies reported the prompt analgesic action of bioactive compounds shortly after CFA-induction. One such case is the flavonoid Luteoloside, which elicited a rapid antinociceptive effect following the first dose and sustained analgesic activity for 14 days [58]. BET is bioaccessible/bioavailable [25], and pharmacokinetic studies have reported systemic absorption and metabolic transformation of BET and related betalains following administration [59,60,61], showing promise for therapeutic use.

CFA injection leads to pain induction by causing tissue damage and triggering the production of inflammatory mediators that mediate the activation of pain nociceptors, promote pain signal transmission to the spinal cord and brain, and recruit immune cells to the site of inflammation [62,63]. Inflammatory mediators such as IL-4, IL-1β, and TNF-α were assessed in our study. Compared with the CFA-treated group, BET treatment increased the anti-inflammatory cytokine IL-4 and reduced proinflammatory IL-1β in a dose-dependent manner, with significant effects at 80 mg/kg, while TNF-α showed a non-significant decrease at both 40 mg/kg and 80 mg/kg. These findings confirm the anti-inflammatory effect of BET and its associated analgesic effect. In fact, BET is able to reprogram microglia from a pro-inflammatory M1 phenotype to an anti-inflammatory M2 phenotype [64]. BET treatment diminished microglial activation in the spinal cord of mice model of neuropathic pain made by CCI, which likely reduced the associated inflammation [65]. Martinez et al. revealed the anti-inflammatory activity of betalain-rich dye in varied models of pain through mechanisms involving the reduction in IL-1β and TNF-α levels [66]. Furthermore, He et al. reported that BET attenuated joint inflammation and reduced cytokine levels through suppression of the MAPK/NF-κB pathway, hence reinforcing the view that BET also exerts such effects in CFA-injected rats [67].

Moreover, oxidative stress parameters like MDA increase following CFA induction and contribute to nociception and inflammation [68,69]. Our results showed that BET decreased the levels of MDA in LSPC in comparison with the CFA-treated rats. Consistent with our findings, recent investigations similarly have also reported that lowering elevated spinal cord MDA levels correlates with reduced hyperalgesia and inflammation [70,71]. The anti-nociceptive effect of BET, in part, stems from its ability to inhibit lipid oxidative damage, as reflected by the reduction in MDA levels. Thus, BET’s ability to lower MDA underscores a potential role in mitigating oxidative stress-driven nociceptive signaling.

Following the construction of miR-107 and miR-145 correlation to biochemical markers; miR-107/MDA, miR-107/IL-1β, and miR-107/TNF-α pairs manifested positive correlation, with anti-correlation in the miR-107/IL-4 pair, while miR-145/IL-1β was negatively correlated and the miR-145/IL-4 pair showed positive correlation. In addition, biplot PCA distinctly segregated the experimental groups, with MDA, IL-1β, TNF-α, and miR-107 variables related to CFA, whereas IL-4 and miR-145 were associated with the control and BET-treated groups. These results point to a possible role of miR-107 and miR-145 in the modulation of inflammatory pain by BET.

Our data demonstrated that miR-107 was upregulated in the LSPC in the CFA-induced inflammatory pain model. Overexpression of miR-107 has been identified in inflammatory diseases. Bioinformatics data on osteoarthritis patients demonstrated miR-107 as a regulator of CFS1 gene encoding for mononuclear phagocytes differentiation [72]. It is noteworthy that miR-107 contributes to redox regulation, as its inhibition was found to upregulate CAB39, thereby activating the AMPK-Nrf2 antioxidant signaling cascade and protecting osteoblasts from dexamethasone-induced oxidative injury [14]. Also, suppression of miR-107 decreases TNF-α secretion in circulating endothelial cells of septic acute renal injury patients [73]. Importantly, it was previously proven that miR-107 underlies inflammatory pain via suppression of GLT-1 expression, leading to reduced glutamate uptake and consequent glutamate accumulation in the rat spinal cord [12]. Such glutamate excess can disrupt calcium homeostasis, triggering oxidative stress and the release of pro-inflammatory cytokines such as IL-1β and TNF-α [74,75]. In addition, these inflammatory cytokines themselves can further impair GLT-1, forming a vicious cycle of transporter failure and inflammation [76,77,78]. Thus, we speculated that the observed downregulation of miR-107 following BET treatment might help to preserve GLT-1 function and activate antioxidant defense pathways. This, in turn, can reduce excitotoxicity and oxidative stress, with immune response modulation.

While there is no literature that investigated miR-145 effect in CFA-induced inflammatory pain, miR-145 has been shown in several recent investigations to suppress pro-inflammatory cytokine release and reduce oxidative stress. For example, in a neuropathic pain rat model induced by CCI, overexpression of miR-145 attenuated mechanical allodynia and thermal hyperalgesia, and notably downregulated IL-1β, TNF-α, and IL-6 via suppression of Akt/mTOR and NF-κB signaling [13]. In cell models, miR-145 overexpression protected cardiomyocytes under high-glucose conditions by decreasing MDA, ROS, IL-6, TNF-α and increasing antioxidant enzyme activities, largely through targeting ARF6 [15]. Another study in myocardial ischemia–reperfusion also showed that miR-145-5p mimics reduced MDA, ROS, IL-1β, and TNF-α under stress [79]. Consistently, our findings show that BET treatment is accompanied by elevated expression of miR-145 in the rat LSPC, suggesting a likely role in controlling inflammatory signaling and oxidative stress alterations in CFA-induced inflammatory pain.

Building on the docking findings, the results suggest that BET may exert its therapeutic effects through multi-target molecular interactions involving both inflammatory signaling pathways and apoptotic regulators. In our study, docking analysis demonstrated that BET exhibited stable interactions with AKT1, mTOR, and IKKβ, which are key components of a well-established inflammatory signaling cascade. Mechanistically, AKT activates IKKβ, which in turn activates the NF-κB signaling pathway, a central transcriptional regulator of inflammation [80,81]. Activated NF-κB promotes the expression of pro-inflammatory cytokines such as TNF-α and IL-1β, as well as enzymes like COX-2, thereby amplifying the inflammatory response [82]. TNF-α is a central pro-inflammatory cytokine that contributes to the release of downstream mediators such as IL-1β and IL-6, as well as nociceptor sensitization and mechanical hyperalgesia. Previous studies have shown that blocking TNF-α in CFA-induced inflammatory models reduces mechanical hyperalgesia [83,84]. Similarly, IL-1β and COX-2 play a crucial role in inflammatory pain signaling by driving peripheral as well as central sensitization [85,86,87]. In parallel, AKT also activates mTOR, a key regulator in pain modulation and inflammation, particularly in dorsal horn neurons, and inhibition of mTOR attenuates mechanical allodynia and thermal hyperalgesia [88,89,90]. Within this framework, the docking results support the interpretation that BET interacts with upstream regulators of inflammation (AKT, mTOR, IKKβ), leading to downstream suppression of inflammatory mediators. This multi-level targeting is particularly relevant in inflammatory pain, where dysregulation of the AKT1–IKKβ–NF-κB axis leads to sustained cytokine production and nociceptor sensitization [91,92]. The ability of BET to interact with both upstream signaling kinases and downstream cytokines suggests a coordinated regulatory effect rather than a single-target mechanism.

In addition to its anti-inflammatory activity, BET is also suggested to exert cytoprotective effects through modulation of apoptotic pathways. The docking analysis revealed that BET interacts with key apoptotic mediators, including caspase-3, -7, and -8. These caspases are central components of the apoptotic cascade, where caspase-8 functions as an initiator in the extrinsic pathway, while caspase-3 and caspase-7 act as executioner caspases responsible for cellular dismantling [93]. Importantly, inflammation and apoptosis are tightly interconnected processes, and dysregulation of NF-κB signaling has been shown to influence both inflammatory responses and apoptotic pathways [94,95]. Based on these findings, it can be proposed that BET could exert cytoprotective effects through modulation of apoptotic executioners. This effect is particularly relevant in inflammatory pain conditions, where excessive inflammation can trigger apoptosis in neuronal and immune cells, contributing to tissue damage and sensitization. By interacting with caspase targets, BET is likely to maintain cellular integrity and reduce apoptosis-associated damage, thereby complementing its anti-inflammatory effects.

Collectively, these findings support a dual-axis mechanism underlying the analgesic activity of BET. First, BET may attenuate inflammation by modulating upstream signaling pathways involving AKT, mTOR, and IKKβ, leading to reduced activation of NF-κB and subsequent suppression of pro-inflammatory mediators such as TNF-α, IL-1β, and COX-2. Second, BET is proposed to exert cytoprotective effects through modulation of apoptotic regulators, including caspase-3, -7, and -8. This integrated mechanism highlights the potential of BET as a multi-target agent capable of simultaneously regulating inflammation and apoptosis, which are key processes underlying inflammatory pain. However, it should be emphasized that the docking results should be interpreted as exploratory and represent predictive insights into potential molecular interactions rather than definitive evidence of direct target engagement in vivo. Thus, further experimental validation using biochemical assays and molecular studies, particularly focusing on AKT/mTOR, NF-κB signaling, and apoptotic pathways, is required to confirm these proposed mechanisms and fully elucidate the therapeutic potential of BET.

Meanwhile, in the present study, ultrastructural examination of the LSPC provided supportive morphological observations consistent with the neuroprotective effects of BET. Neurons from CFA-treated rats exhibited marked structural abnormalities, including chromatin condensation, nuclear blebbing, and swollen mitochondria. Previous studies have shown that proinflammatory cytokines can directly impair the mitochondrial membrane potential, thereby facilitating cytochrome c release and caspase-dependent apoptosis in spinal neurons [96,97,98]. In parallel, ROS-induced oxidative stress further destabilizes mitochondrial membranes [99,100,101], exacerbating neuronal injury and central sensitization. Therefore, the ultrastructural improvements in nuclear morphology and mitochondrial integrity after BET treatment are likely attributable to its ability to reduce neuroinflammatory burden and limit oxidative damage within the LSPC microenvironment. The greater protection observed at 80 mg/kg aligns with the stronger suppression of IL-1β and MDA at this dose. Furthermore, the concurrent increase in IL-4 may have contributed to neuronal preservation by inhibiting glial overactivation and enhancing anti-inflammatory signaling pathways known to support cellular survival and tissue repair [102]. Moreover, the predicted interaction of BET with caspase-3, -7, and -8 suggests a potential anti-apoptotic contribution to its neuroprotective profile. Experimental evidence indicates that blockage of these caspases by natural compounds can reduce neuronal degeneration in models of neuroinflammation and pain [103,104,105].

Despite this study providing some valuable perceptions into the anti-nociceptive, anti-inflammatory, and antioxidant effects of BET, it has some limitations. First, the sample size per experimental group was relatively small, which might reduce statistical power in some instances and warrants validation in larger cohorts in future studies. Second, the absence of a reference (positive control) drug limits the ability to directly compare BET’s analgesic potency with established treatments. Third, there was a lack of a wider dose range of BET to better define its optimal therapeutic dose. Fourth, mechanical allodynia was not assessed, and therefore our findings are not comparable with previous reports.

## 5. Conclusions

The present study provides preliminary evidence that BET could relieve CFA-induced inflammatory pain through analgesic, anti-inflammatory, and antioxidant activities, as reflected by improvements in paw edema, thermal nociceptive response, oxidative stress markers, and inflammatory cytokine profiles. These actions are hypothesized to be linked to miR-107 downregulation and miR-145 upregulation in parallel with changes in MDA, IL-4, IL-1β, and TNF-α levels. While these associations suggest a potential role of BET in supporting the maintenance of redox homeostasis and promotion of an anti-inflammatory immunological milieu, further validation is required to establish a definitive causal mechanism. Molecular docking results further suggested in silico evidence of interactions of BET with key inflammatory and apoptotic targets, highlighting a plausible route through which BET may modulate inflammatory and apoptotic signaling. Collectively, these findings support BET as a promising natural bioactive compound capable of alleviating inflammatory pain and counteracting oxidative and cytokine dysregulations, offering a foundation for developing novel, plant-derived analgesic therapies.

## Figures and Tables

**Table 1:** List of miRNAs, GeneGlobe Assay IDs, and Suppliers.

miRNA	GeneGlobe Assay ID	Supplier
miR-107	YP00204468	Qiagen
miR-145	YP00204483	Qiagen
miR-103-3p (control)	YP00204063	Qiagen

**Table 2:** The correlation matrix between the investigated biomarkers.

		miR145	miR107	MDA	IL-4	IL-1ß
miR107	Pearson Correlation	−0.406				
	Sig. (2-tailed)	0.191				
MDA	Pearson Correlation	−0.416	0.840 **			
	Sig. (2-tailed)	0.178	0.001			
IL-4	Pearson Correlation	0.585 *	−0.908 **	−0.864 **		
	Sig. (2-tailed)	0.045	0.000	0.000		
IL-1ß	Pearson Correlation	−0.634 *	0.591 *	0.728 **	−0.608 *	
	Sig. (2-tailed)	0.027	0.043	0.007	0.036	
TNF-α	Pearson Correlation	−0.465	0.686 *	0.832 **	−0.662 *	0.932 **
	Sig. (2-tailed)	0.128	0.014	0.001	0.019	0.000

*. Correlation is significant at the 0.05 level (2-tailed). **. Correlation is significant at the 0.01 level (2-tailed).

**Table 3:** Docking results and validation parameters.

Protein	BET Binding Affinity (kcal/mol)	Co-Crystallized Ligand	Native Ligand Binding Affinity (kcal/mol)	RMSD (Å)
AKT1	−9.0	0XZ	−8.7	0.4752
mTOR Kinase	−7.6	17G	−11.5	0.5351
IKKβ	−9.2	KSA	−11.7	1.0819
TNF-α	−8.2	SPD304	−9.1	1.0065
IL-1β	−6.7	T9C	−8.7	0.6231
COX-2	−8.7	ID8	−8.6	0.6554
Caspase-3	−8.4	B92	−8.0	1.4612
Caspase-7	−7.5	NXN	−7.1	0.7497
Caspase-8	−7.1	B94	−6.5	1.4270

**Table 4:** Molecular interactions between BET and AKT1, mTOR, IKKβ, TNF-α, IL-1β, COX-2, caspase-3, caspase-7, and caspase-8.

BET/Receptor	Interacting Residues	Ligand Atom	Bond Distance (Å)	Interaction
AKT1	GLY159:HN (A), LYS276:HZ1 (A),	BET:O	3.08, 2.18	H-Bond
	LEU156:O (A), ASN279:OD1 (A), GLY311:O (A), ASP274:OD2 (A)	BET:H	2.46, 2.97, 2.26, 1.88	
	ASP292:OD1 (A)	BET:Ring	4.12	Pi-Anion
mTOR Kinase	VAL2240:HN (B)	BET: OXT	1.89	H-Bond
	THR2245:HN (B), ARG2251:HH22 (B)	BET:O	2.62, 2.66	
	ASP2252:OD1 (B), CYS2243:O (B), CYS2243:O (B)	BET:H	2.42, 2.58, 2.25	
IKKβ	TYR169:HH (B)	BET:OXT	2.37	H-Bond
	GLU149:O (B), ASP145:OD1 (B), CYS99:O (B), GLU97:O (B)	BET:H	2.26, 2.01, 2.88, 1.76	
	VAL29:CG2 (B), ILE165:CD1 (B)	BET:Ring	3.58, 3.75	Pi-Sigma
TNF-α	SER60:O (A), TYR119:O (B), SER95:O (B)	BET:H	2.19, 2.71, 2.43	H-Bond
	LEU120:HN (A), GLN125:HE22 (D)	BET:O	2.54, 2.59	
	GLY121:HN (A)	BET:OXT	2.88	
	LEU55:CD2 (D)	BET:Ring	3.73	Pi–Sigma
IL-1β	LYS55:HZ1 (A), LYS55:HZ2 (A), ASN102:HD21 (A)	BET:OXT	2.59, 2.73, 2.40	H-Bond
	SER45:O (A)	BET:H	2.45	
	LYS94:CA (A)	BET:O	3.55	
	PRO57 (A), VAL100 (A), ALA115 (A)	BET:Ring	4.71, 4.62, 5.28	Pi-Alkyl
COX-2	PHE580:HN (A), HIS90:CE1 (A), GLY354:CA (A)	BET:O	2.07, 3.50, 3.42	H-Bond
	TYR355:O (A), GLN192:O (A), PRO514:O (A)	BET:H	2.54, 2.78, 1.94	
Caspase-3	HIS121:HD1 (A)	BET:N	2.32	H-Bond
	HIS121:HD1 (A), ARG207:HE (B)	BET:OXT	2.34, 2.32	
	HIS121:HD1 (A), PHE250:HN (B), SER249:CA (B)	BET:O	2.85, 2.08, 3.51	
	GLU248:O (B)	BET:C	3.40	
	TRP206 (B)	BET:Ring	5.25	Pi-Pi T-shaped
Caspase-7	ARG187:HH11 (A), ARG187:HH12 (A), THR225:HN (A), THR225:HG1 (A)	BET:O	3.01, 2.87, 2.86, 2.00	H-Bond
	A:LYS212:O (A), GLU147:OE1(A), GLU147:O (A), PHE221 (A)	BET:H	3.06, 2.77, 2.03, 2.75	
	GLN287:HE22 (A)	BET:OXT	2.83	
	TYR223 (A)	BET:C	3.59	Pi-Sigma
	CYS290:SG (A)	BET:Ring	5.05	Pi-Sulfur
	TYR211 (A), TYR223 (A)	BET:Ring	4.66, 3.98	Pi-Pi Stacked
Caspase-8	ILE333:HN (A), TYR334:HN (A), THR467:HG1 (B)	BET:O	2.80, 1.93, 2.07	H-Bond
	GLN465:HE21 (B)	BET:N	2.88	
	TYR334:OH (A)	BET:H	2.40	
	LYS320:O (A)	BET:C	3.16	
	LYS320:C,O;GLY321:N (A)	BET:Ring	4.52	Amide-Pi Stacked

CD1 and CD2 denote side-chain carbon atoms (e.g., in leucine); HE21 and HE22 represent amide hydrogens in glutamine; HH denotes a hydroxyl hydrogen (e.g., in tyrosine); HH11 and HH12 denote guanidinium hydrogens in arginine; HZ1 and HZ2 represent nitrogen-bound side-chain hydrogens in lysine; HD21 and HD22 are amide hydrogens in asparagine; OD1/OD2 and OE1/OE2 denote side-chain carboxylate oxygens in aspartate and glutamate, respectively; ND2 represents the amide nitrogen in asparagine; HG1 and HD1 denote side-chain hydrogens at the gamma and delta positions, respectively; CE1 represents an epsilon-position carbon (typically in aromatic residues); SG denotes a sulfur atom at the gamma position (e.g., in cysteine); OXT denotes a terminal oxygen atom; HN is the backbone amide hydrogen; CA is the alpha carbon.

## Data Availability

The raw data supporting the conclusions of this article will be made available by the authors on request.

## Supplementary Materials

The following supporting information can be downloaded at: https://www.mdpi.com/article/10.3390/biomedicines14061202/s1, File S1: Detailed Molecular Docking Protocol; Table S1: PDB IDs, co-crystallized ligands, and grid box parameters used for molecular docking. References [106,107,108] are cited in the Supplementary Materials.

## Abbreviations

The following abbreviations are used in this manuscript:

AKT1	RAC-alpha serine/threonine-protein kinase
AMPK	AMP-activated protein kinase
ANOVA	Analysis of Variance
ARF6	ADP-Ribosylation Factor 6
BET	Betanin
BFGS	Broyden–Fletcher–Goldfarb–Shanno method
CAB39	Calcium Binding Protein 39
CCI	Chronic Constriction Injury
cDNA	Complementary DNA
CFA	Complete Freund’s Adjuvant
COX-2	Cyclooxygenase-2
CSF1	Colony-Stimulating Factor 1
GLT-1	Glutamate Transporter 1
HPL	Hot Plate Latency
IKKβ	Inhibitory kappa B kinase beta
IL-1β	Interleukin-1 beta
IL-4	Interleukin-4
LPO	Lipid Peroxidation
LSD	Least Significant Difference
LSPC	Lumbar Spinal Cord
MDA	Malondialdehyde
miR-107	microRNA-107
miR-145	microRNA-145
mTOR	Mammalian Target of Rapamycin
NF-κB	Nuclear Factor kappa B
Nrf2	Nuclear factor erythroid 2-related factor 2
NSAIDs	Nonsteroidal Anti-Inflammatory Drugs
PBS	Phosphate-Buffered Saline
PCA	Principal Component Analysis
PDB	Protein Data Bank
qRT–PCR	Quantitative Reverse Transcription Polymerase Chain Reaction
RMSD	Root Mean Square Deviation
ROS	Reactive Oxygen Species
TBA	Thiobarbituric Acid
TBARS	Thiobarbituric Acid Reactive Substances
TEM	Transmission Electron Microscopy
TNF-α	Tumor Necrosis Factor alpha
