# Supplementary material for: Betanin from Beta vulgaris Attenuates Complete Freund’s Adjuvant-Induced Inflammatory Pain: Integrated Preclinical and In Silico Insights
Source: Biomedicines. 2026 May 27;14(6):1202. doi: 10.3390/biomedicines14061202 (PMC13296267; doi:10.3390/biomedicines14061202)
Supplement: Supplementary file 1 [file biomedicines-14-01202-s001.zip › biomedicines-4296449-supplementary.pdf]

Article

Betanin from *Beta vulgaris* Attenuates Complete Freund's Adjuvant-Induced Inflammatory Pain: Integrated Preclinical and In Silico Insights

Ahmed Massoud [1,2,*], Amina E. Essawy [1], Mohammed A. Alfredan [3], Ashraf M. Abdel-Moneim [1,3], Rehab A. Gomaa [1] and Sherine Abdel Salam [1,3,*],

[1] Department of Zoology, Faculty of Science, Alexandria University, Alexandria 21511, Egypt
[2] Faculty of Science, Alamein International University, New Alamein City 51718, Egypt
[3] Department of Biological Sciences, Faculty of Science, King Faisal University, Al-Ahsa 31982, Saudi Arabia
* Correspondence: amasoud97@hotmail.com (A.M.); sherine.abdel.salam@alexu.edu.eg (S.A.S.)

Supplementary Material

Supplementary Method S1: Detailed Molecular Docking Protocol

Protein and Ligand Preparation

Prior to molecular docking, protein preparation was carried out using AutoDock Tools version 1.5.7 [1,2]. All crystallographic water molecules were removed to avoid potential interference with ligand binding. Polar hydrogen atoms were added, Kollman charges were assigned, and non-polar hydrogens were merged to simplify the structure. The finalized protein models were saved in PDBQT format. For the ligand, the three-dimensional structure of BET was geometry-optimized and energy-minimized using Avogadro version 1.2.0 with the MMFF94 force field to ensure a stable conformation [3]. The ligand was then processed in AutoDock Tools 1.5.7, where Gasteiger charges were assigned, non-polar hydrogens were merged, and rotatable bonds were defined before being saved in PDBQT format.

Docking Parameters

The search exhaustiveness was set to 8 to balance accuracy and computational efficiency. The docking grid boxes for all target proteins were centered on the coordinates of their co-crystallized ligands. The corresponding grid box parameters are detailed in Supplementary Table S1.

Supplementary Table S1. PDB IDs, co-crystallized ligands, and grid box parameters used for molecular docking.

Protein	PDB ID	Co-crystallized Ligand	Center (x, y, z)	Size (Å)
AKT1	4GV1	0XZ	-20.005, 4.344, 10.739	36×36×36
mTOR Kinase	4JSX	17G	–19.697, –32.338, –56.084	30×30×30
IKKβ	4KIK	KSA	-13.758, -32.094, -73.614	34×34×34
TNF-α	2AZ5	SPD304	–19.409, 74.651, 33.849	30×28×30
IL-1β	8C3U	T9C	–18.083, –5.039, –41.894	36×36×34
COX-2	5IKR	ID8	38.961, 2.353, 61.503	34×34×34
Caspase-3	3KJF	B92	21.578, -5.202, 10.866	38×36×36
Caspase-7	1SHJ	NXN	52.084, 16.917, 1.252	28×28×28
Caspase-8	3KJQ	B94	-11.011, 34.519, 15.723	28×28×28

References

1. Goodsell, D.S.; Olson, A.J. Automated docking of substrates to proteins by simulated annealing. *Proteins* **1990**, *8*, 195–202, doi:10.1002/prot.340080302.
2. Morris, G.M.; Huey, R.; Lindstrom, W.; Sanner, M.F.; Belew, R.K.; Goodsell, D.S.; Olson, A.J. AutoDock4 and AutoDockTools4: Automated docking with selective receptor flexibility. *J. Comput. Chem.* **2009**, *30*, 2785–2791, doi:10.1002/jcc.21256.
3. Hanwell, M.D.; Curtis, D.E.; Lonie, D.C.; Vandermeersch, T.; Zurek, E.; Hutchison, G.R. Avogadro: An Advanced Semantic Chemical Editor, Visualization, and Analysis Platform. *J. Cheminform.* **2012**, *4*, 17, doi:10.1186/1758-2946-4-17.
